# Comparison of Serum and Urine Neutrophil Gelatinase-Associated Lipocalin (NGAL) With Serum Creatinine in Prediction of Kidney Suitability for Transplantation

**DOI:** 10.5812/numonthly.5002

**Published:** 2012-12-15

**Authors:** Mitra Mahdavi-Mazdeh, Alireza Abdollahi, Behnaz Nozary Heshmati, Zahra Sobhani

**Affiliations:** 1 Iranian Tissue Bank and Research Center, Tehran University of Medical Sciences, Tehran, IR Iran; 2 Research Center of Nephrology, Tehran University of Medical Sciences, Tehran, IR Iran

**Keywords:** NGAL, Tissue Donors, Kidney Transplantation

## Abstract

**Background:**

When using brain dead donors for organ donation, assessment of kidney function before the procedure is essential.

**Objectives:**

It would be ideal to find an early marker of viability of donor kidneys that is more accurate than creatinine.

**Patients and Methods:**

The present study was conducted from March of 2011 to March of 2012, included 52 deceased donors. Serum and urinary Neutrophil Gelatinase Associated Lipocalin (NGAL) and creatinine were measured at 0, 2, 4, 8, 12 and 18 hours after their admission to the ICU of our organ procurement unit (OPU). Other routine laboratory tests of hemglobin, blood glucose and lipid profile were performed as well.

**Results:**

There were 31 males and 21 females with the mean age of 29.7 ± 14.3 (6-58) years. Thirty one patients became brain dead due to car accidents. The mean time of admission time before harvest was 12.6 ± 5.8 (3-30) hours. We did not discover any correlation of serum creatinine with serum or urinary NGAL at any time before the organ harvesting. However, serum NGAL level measurement 2 hours after admission correlated significantly with other hours' measurements (4, 6, 8 hours, r > 0.9; *P* < 0.001).

**Conclusions:**

The serum NGAL level, especially at 2 hours after admission to the ICU, should be evaluated with kidney function after transplantation to get the accurate predictive value.

## 1. Background

Acute Kidney Injury is a common problem in many clinical settings including the complex process of organ retrieval from brain dead donors (BDD). Currently, the markers for early diagnosis of deteriorating renal function such as serum creatinine levels or urinary output are not very reliable, and have many limitations. A rise in creatinine is a late sign of kidney damage; therefore it cannot be a reliable indicator of acute changes in kidney function. Discovery of a predictor biomarker of acute kidney injury would be of great value.

Neutrophil gelatinase-associated lipocalin (NGAL) is a small size molecule (25 kDa) that is resistant to degradation. Belonging to the lipocalin superfamily, it was initially found in activated neutrophils. It can be easily excreted in urine. Moreover, its enhanced excretion may be due to early tubular cell injury and failure to reabsorb the freely filtered NGAL load (2, 3). However, renal tubular cells may release NGAL in response to inflammation or injury. Urinary NGAL can be an early predictive biomarker of delayed graft function due to ischemia-reperfusion injury (2, 4). Until brain death, the donor is at risk of incurring different insults, for example, sepsis, blood pressure fluctuation and electrolyte imbalances. Assessment of the medical suitability of the donor and kidney quality before transplantation is the major responsibility of the organ procurement organizations (OPU) (5).

In the meta-analysis of data from 19 studies, involving 2,538 patients, it has been well documented that urine, and /or plasma NGAL level increase, preceded a clinical diagnosis of acute kidney injury and it has prognostic significance. The similar diagnostic accuracy of plasma/serum NGAL (17.9 [95% CI, 6.0-53.7]/0.775 [95% CI, 0.679-0.869]) to that of urine NGAL (18.6 [95% CI, 7.2-48.4]/0.837 [95% CI, 0.762-0.906]) was also shown within 6 hours from the time of insult (if known) or 24-48 hours before the diagnosis of AKI if the time of insult was not known. The cutoff of NGAL concentration for most favorable sensitivity and specificity to predict AKI across all settings ranged from 100-270 ng/mL, with higher values for adults (170 ng/mL) in comparison with children (100-135 ng/mL) (6). However, Bahngoo *et al*. could not find reliable studies in their meta-analysis for urine NGAL levels and suggested that higher quality studies needed to be done to validate novel biomarkers in larger donor populations (5).

Mishra *et al*. examined 25 specimens of kidney biopsies taken at approximately one hour of reperfusion after transplantation by immunohistochemistry and found the strong correlation of NGAL expression not only with cold ischemia time (R = 0.87, *P* < 0.001) but also with peak postoperative creatinine level days later (R = 0.86, *P* < 0.001) (7). Kuska *et al*. highlighted the value of serum NGAL level in prediction of early functional recovery of transplanted kidneys from cardiac dead donors compared with living donors (8).

## 2. Objectives

Currently, those donors with normal serum creatinine and urine output are known as suitable kidney donors. Our objective was to investigate whether serum and urine NGAL level has the predictive value for kidney suitability for retrieval and transplantation. This study also addresses the best time for NGAL measurement in deceased donors.

## 3. Patients and Methods

The present study included 52 patients who were recognized as deceased donors from March of 2011 to March of 2012 at our center. The protocol was approved by the ethics committee of Tehran university of medical sciences. Serum NGAL (Antibody shop, Gentofte, Denmark) and creatinine, hemglobin, blood glucose (commercially available kits) were measured at 0, 2, 4, 8, 12 and 18 hours after admission to ICU to our organ procurement unit (OPU). Serum creatinine was measured by Jaffe´s method. All measurements were performed by personnel for whom patient information was unknown. Samples were centrifuged at 2,500 rpm at 4°C for 10 minutes, and then the supernatants of serum and urine were frozen at 70°C. Other routine laboratory tests of hemglobin, blood glucose and lipid profile were also performed.

Data were expressed as mean ± standard deviations or medians (minimum, maximum values). The examination of the distribution normality of variables was performed using W-Shapiro-Wilk test. Correlations between NGAL and other variables were evaluated by Pearson’s or Spearman’s test as appropriate. *P* < 0.05 has been considered significant.

## 4. Results

The studied population was 52 patients. There were 31 male and 21 females. The mean age was 29.7 ± 14.3 (6-58) years. The median of donors' age was 28 years. Thirty one became brain dead due to car accidents. The mean time of admission time before harvest was 12.6 ± 5.8 (3-30) hours, with the median of 12.5 hours. The mean and median of serum creatinine were 1.17 ± 0.4 mg/dL and 1.1 mg/dL respectively. The demographic data of patients are presented in Table 1.

**Table 1:** Demographic Data of Brain Dead Population Admitted to the Center

Characteristics	Mean ± SD (Min-Max)
**Age, y**	29.7 ± 14.3 (6-58)
**Sex, M/F**	31/21
**Systolic BP0, CmHg**	12.1 ± 2.3 (5.2-16.3)
**Diastolic BP0, CmHg**	7.7 ± 1.7 (3.0-12.0)
**Hemoglobin, g/dL**	12.4 ± 2.7 (7.5-19.5)-51
**Creatinine, mg/dL**	1.14 ± 0.4 (0.5-2.7)
**Cholesterol, mg/dL**	141.2 ± 41.8 (71-254)
**Triglyceride, mg/dL**	111.3 ± 51.8 (36-280)
**Mean serum NGAL, ng/mL**	403.5 ± 313.8 (86-1722)
**Mean urine NGAL, ng/mL**	108.3 ± 165.9 (5-655)
**Blood sugar, mg/dL**	187.4 ± 117.7 (55-566)
**Harvesting time, h**	12.6 ± 5.8 (3.0-30.0)

Abbreviations: F; female, M; male

We did not find any correlation of serum creatinine before organ recovery with either serum or urinary NGAL or with mean of serum or urine NGAL levels. However, serum NGAl level measurement after 2 hours correlated significantly with other time point measurements of serum NGAL (4, 6, 8 hours, r > 0.9; *P* < 0.001).

Mean serum and urine NGAL level did not show significant differences between sex of donors or history of hypertension and also did not show any correlation with duration of patient stay in ICU before harvesting time or hourly urine output. However, mean urine NGAL level correlated significantly with hourly urine output (r = 0.38; *P* = 0.015) (Table 2).

**Table 2:** Comparison of Serum and Urinary NGAL With Serum Creatinine in Different Hours of Measurement Prior Organ Retrieval

Time (h)	Serum Cr Mean ± SD (min-Max)	Serum NGAL Mean ± SD (min-Max)	Urinary NGAL Mean ± SD (min-Max)	Serum NGAL/Crea Mean ± SD (min-Max)	Urinary NGAL/Crea Mean ± SD (min-Max)
**0**	1.38 ± 0.70 (0.4-2.6)	428.9 ± 276.6 (90-1360)	119.7 ± 230.1 (5-1000)	315.6 ± 236.2 (38-850)	18.4 ± 45.9 (0.1-175)
**2**	1.30 ± 0.66 (0.5-2.4)	390.0 ± 340.7 (80-2000)	102.4 ± 179.1 (5-945)	253.4 ± 167.0 (88.9-733.3)	39.7 ± 104.0 (2.3-400)
**4**	1.24 ± 0.50 (0.5-2.2)	383.6 ± 361.2 (80-1900)	115.0 ± 212.8 (5-960)	288.8 ± 166.9 (52.9-628.6)	19.1 ± 47.0 (0.1-180.4)
**8**	1.33 ± 0.73 (0.5-2.6)	394.6 ± 449.0 (90-1910)	89.7 ± 162.2 (5-600)	235.2 ± 185.7 (34.6-466.7)	1.3 ± 2.4 (0.1-4.44)
**12**	1.10 ± 0.43 (0.5-1.8)	232.5 ± 160.3 (70-600)	101.7 ± 150.0 (5-445)	241.6 ± 229.4 (68.8-620.0)	0.7 ± 1.2 (0.1-6.2)
**18**	1.08 ± 0.43 (0.6-1.07)	246.7 ± 127.5 (90-440)	42.8 ± 82.6 (5-260)	280.6 ± 3.9 (277.8-283.3)	0.5 ± 0.4 (0.1-0.9)

Abbreviations: NGAL; neutrophil gelatinase-associated lipocalin

## 5. Discussion

Delayed graft function (DGF) is a common complication in transplanted patients from deceased donors. Hollmen *et al*. in the study on 99 brain dead donors and their 176 adult recipients showed that kidneys from donors with urine NGAL levels higher than the mean (≥ 18 ng/mL) were more likely to show prolonged delayed graft function (DGF), and emphasized its value as an independent risk factor of DGF but they could not show a difference of donor Serum NGAL between those with prolonged, short DGF and early graft function (9). In the study of Junge *et al*. in 2006 the correlation of urinary and serum NGAL values with consequent graft function in 30 brain dead kidney donors was revealed and the predictive value for the urinary NGAL level (below 65 ng/mL) were associated with immediate graft function and levels above 150 ng/mL were associated with delayed or primary non-function of grafts (1). In another study of Hollmen *et al*. on 176 kidney recipients with deceased donor kidneys, it showed that urinary NGAL levels (before and days after transplantation) decreased more slowly in those with DGF compared with those without DGF (4). In our investigation, however, serum and urinary NGAL levels did not show a significant correlation with serum creatinine before organ retrieval. It was also shown that the level did not have correlation with age or other routine laboratory data. However, in our study, the mean serum NGAL level of patients was higher than normal (428.9 ± 276.6ng/mL), although the serum creatinine was in low normal range. It means that there are some other subtle injuries in the kidney that cannot be certified with creatinine level and we have to look for these changes by studying the correlation of serum/urine NGAL level with graft function. In our opinion, the main reason for this was the characteristics of Iranian brain dead donors. In general, they were young and did not experience significant hypotensive periods (the therapeutic goal was systolic blood pressure, 100 mmHg), oliguria (urine output < 20 mL/h), or hypothermia (10, 11). Consequently, it is possible that the damage to the kidney needs to be followed until after the kidney has been transplanted. In conclusion, it seems that this study needs to be continued to see the predictive value of high level of urine and serum NGAL levels by the current golden standard of donated kidney suitability of graft function.
